# The prebiotic and anti-fatigue effects of hyaluronan

**DOI:** 10.3389/fnut.2022.977556

**Published:** 2022-08-08

**Authors:** Guoxin Huang, Lu Su, Ni Zhang, Ruixuan Han, Wai Kit Leong, Xiaoang Li, Xuecong Ren, W. L. Wendy Hsiao

**Affiliations:** ^1^State Key Laboratory of Quality Research in Chinese Medicine, Macau University of Science and Technology, Macau, China; ^2^Clinical Research Center, Shantou Central Hospital, Shantou, China; ^3^Zhuhai MUST Science and Technology Research Institute, Zhuhai, China; ^4^Department of Cell Biology, Zhejiang University School of Medicine, Hangzhou, China; ^5^Zhejiang University Medical Center, Hangzhou, China; ^6^Center for Stem Cell and Regenerative Medicine, Zhejiang University School of Medicine, Hangzhou, China; ^7^Foshan Women and Children Hospital Affiliated With Southern Medical University, Foshan, China

**Keywords:** hyaluronan, gut microbiota, anti-fatigue, short-chain fatty acid producer, sulfate-reducing bacteria, mitochondrial toxicity

## Abstract

Hyaluronan (HA) is a mucopolysaccharide that naturally exists in all living organisms as the main component of the extracellular matrix. Over the last 30 years, HA has been used as the main ingredient in cosmetic products, eye drops, and medicinal products. It is also taken orally as a health supplement. However, the physiological effect of the ingested HA is not clear. In the current study, the interaction between HA and gut microbiota, and the potential prebiotic effects were investigated. HA was used to treat the C57BL/6 mice for 15 consecutive days, then fecal genomic DNA was extracted from fecal samples for 16S rRNA amplicon sequencing. The results showed that HA could significantly change the composition of gut microbiota (GM), e.g., increased the relative abundance of beneficial bacteria, including short-chain fatty acids (SCFAs)-producing bacteria and xylan/cellulose-degrading bacteria, whereas decreased the relative abundance of potential pathogens including sulfate-reducing bacteria (SRB), inflammation and cancer-related bacteria. The rotarod test was used to evaluate the anti-fatigue effects of HA in C57BL/6 mice. The results showed that HA could lengthen the mice's retention time on the accelerating rotarod. HA increased the concentration of glycogen and superoxide dismutase (SOD) in mice's muscle and liver, whereas decreased the serum concentration of malondialdehyde (MDA). Moreover, the metabolic products of *Desulfovibrio vulgaris* (MPDV), the model SRB bacteria, showed cytotoxic effects on H9c2 cardiomyocytes in a dosage-dependent manner. MPDV also caused mitochondrial damage by inducing mitochondrial fragmentation, depolarization, and powerless ATP production. Taken together, we show that HA possesses significant prebiotic and anti-fatigue effects in C57BL/6 mice.

## Introduction

Hyaluronan (HA) belongs to the family of mucopolysaccharides (or called glycosaminoglycans) composed of linear polymers of repeating glucuronic acid and N-acetylglucosamine. HA is the main component of extracellular matrix (ECM), which holds the cells together and provides a pathway for the diffusion of nutrients and oxygen to individual cells. It serves as a structural component in different tissues and organs. For example, it serves as lubricants in the synovial fluid of joints, gives jelly-like consistency to the eye and maintains the elastoviscosity of liquid connective tissues, controls tissue hydration and water transport (1, 2). HA also can act as signaling molecules through multiple processes such as morphogenesis, wound healing, inflammation, and cell transformation. Many of the biological activities are through the interaction between HA and its cell surface receptors, CD44, RHAMM, and ICAM-1 (intercellular adhesion molecules-1) (3, 4). HA was also found to enhance peripheral nerve regeneration in the frog (5). In addition, several reports showed that HA possessed free radical scavenging and antioxidant activities *in vivo* and *in vitro* settings (6–8). Reactive oxygen species (ROS) have been proven as endogenous mediators of muscle fatigue and possibly involved in chronic fatigue syndrome (9, 10).

Non-digestible polysaccharides, along with polyphenols, fibers, and plant foods represent a group of prebiotics, which can selectively promote the growth of beneficial bacteria and positively affect the host's physiology (11). Substantial reports showed that polysaccharides from different sources could remarkably change the gut microbiota (GM) composition and improve the gut microenvironment (12–15). As mentioned above, for its unique biological functions, HA can be applied ectopically as skincare and cosmetic products, and eye drops. On the other hand, HA can be taken orally as functional foods for various health benefits. Interestingly, certain bacteria, e.g., *Streptococcus equi* and *Streptococcus zooepidemicus* can synthesize HA (16). Meanwhile, other types of bacteria, for instance, *Staphylococcus aureus* and *Clostridium perfringens* can produce hyaluronidase to degrade HA (17–19). This finding supports the notion that HA could be readily fermented by the host's gut microbes and elicited various biological functions in the host. A piece of good evidence came from a recent report showing HA could alter gut microbiome composition and metabolites in mice, in turn, effectively reducing enteric infection and inflammation (20).

Here, considering the unique physiological features of HA, we propose that some of the functions of HA might be through the modulation of the host's gut microflora. In addition, we also proposed that HA might possess anti-fatigue effects as HA could serve as the source of SCFAs through the fermentation of gut microbes (21). Reports showed that the fatigue indexes (e.g., liver and muscle glycogen) were significantly related to the SCFAs content and the anti-oxidant indexes (e.g., SOD and MDA) (22, 23). To address our hypotheses, C57BL/6 mice were orally administrated with HA for 15 consecutive days. Fecal DNA was extracted for 16s rRNA gene sequencing analysis. The rotarod test and the anti-oxidant indexes (SOD, MDA, and glycogen) in the serum, liver, and muscle were used to evaluate the anti-fatigue effects of HA. Moreover, the mitochondrial morphology, membrane potential, and ATP level were employed to evaluate the effect of metabolic products of *Desulfervibrio*, a group of potentially pathogenic bacteria suppressed by HA treatment.

## Materials and methods

### Animals and treatment

**Figure 1:**
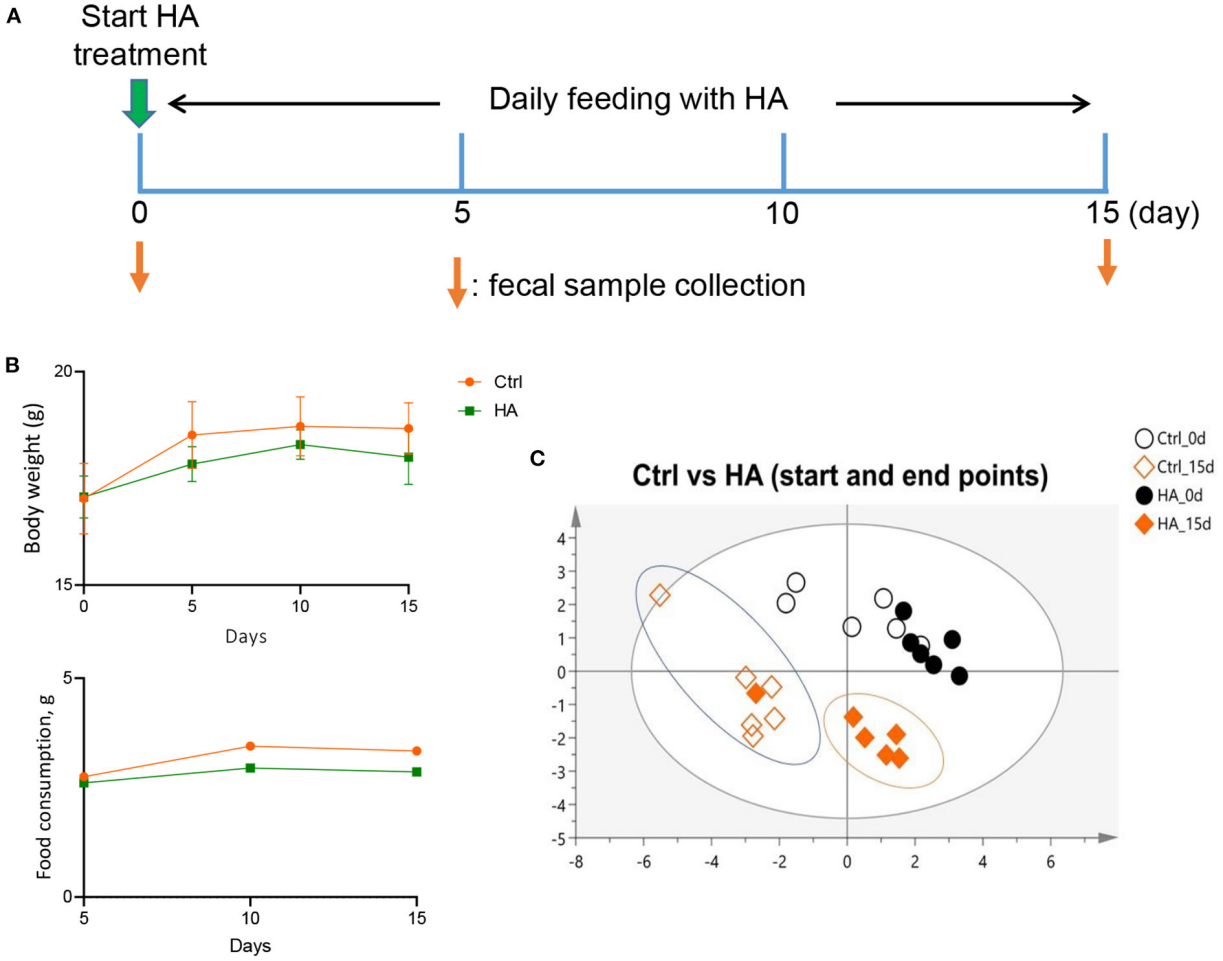
Effect of HA treatment on GM profile of C57BL/6 mice. **(A)** The HA treatment schemes. **(B)** The profiles of food consumption and body weight. **(C)** The similarity of the GM profile of the treatment groups was analyzed by ERIC-PCR and plotted with PLS-DA tool. *n* = 6.

C57BL/6 mice (6–8 weeks old) were purchased from the Chinese University of Hong Kong. The animal welfare and experiments strictly followed the procedures approved by the Ethics Review Committee of Macau University of Science and Technology. The mice were housed in a 12 h dark-light cycle facility and had free access to food (LabDiet, USA) and Milli-Q water. A total of 12 mice were equally divided into 2 groups, i.e., the control group and the HA group. HA was purchased from Aladdin (H107141, Shanghai, China). Mice were daily gavage with 50 mg/kg HA or Milli-Q water for 15 consecutive days. The body weight, food, and water consumption of the mice were recorded every 5 days. Fecal samples were collected individually on 0 and 15 days. All samples were stored at −80°C for later experiments. The experimental scheme was showed in Figure 1A. Mice were anesthetized and sacrificed with pentobarbital sodium.

The rotarod test was performed following our previous study (24). Briefly, mice were subjected to a 1-day learning period at a constant speed of 15 rpm, then scored at an accelerating speed of 20 rpm. The retention time on the rotarod was recorded from three consecutive attempts. The total number of falls from the rotarod within 15 min was recorded for each mouse. The rotarod test was conducted every five days.

### Enterobacterial repetitive intergenic consensus-PCR analysis

The fecal DNA was extracted using QIAamp DNA Stool Mini Kit (QIAGEN, Germany) followed the manufacturer's manual, and analyzed for highly conserved ERIC regions with a pair of ERIC primers sequences: ERIC1 (5′-ATGTAAGCTCCTGGGGATTCAC-3′) and ERIC2 (5′-AAGTAAGTGACTGGGGTGAGCG-3′). PCR conditions were set following the previous description (25). ERIC-PCR products were isolated in 2% agarose gel at 100 V for 45 min. Bands were visualized with the Gel Doc XR+ system and digitized for clustering analysis through SIMCA-P 14.0 tool (Umetrics, Umea, Sweden) with a confidence level 95% (*p* < 0.05).

### 16S rRNA gene sequencing

DNA samples were sequenced for 16S rRNA gene using Illumina MiSeq platform (Illumina, San Diego), targeting the V3–V4 region by barcoded 515F and 806R universal primers and processed as previously described (26). In short, dual-index barcodes and Illumina sequencing adapters were used to join the reads by a limited PCR cycle. After purification with Agencourt AMPure beads (Agencourt, USA), Nextera XT protocol was used for library normalization. And then, samples were loaded into a single flow cell for sequencing on the MiSeq sequencing platform (Illumina, San Diego) following the manufacturer's introduction. Clusters were auto-generated and paired-end sequenced with dual index reads in a single run with a read length of 2 × 300 bp. PANDAseq was used to collect paired end sequences, then Raw FASTQ files were obtained (27). Sequences were trimmed for primers and barcodes. The cleaned sequences were clustered at *k* = 10 (97% similarity) by deletion of chimeras and singleton reads (28, 29). Finally, OTUs were classified using BLASTn against a curated database derived from NCBI.

### SOD, MDA, and glycogen assays

SOD, MDA, and glycogen were tested including the serum, liver, and muscle of the mice. The procedures were carried out according to the manufacturer's manuals (Nanjing Jiancheng Bioengineering Institute, China). SOD was assayed using a hydroxylamine reaction and detected at 550 nm. MDA was determined in a thiobarbituric acid reaction and measured at 532 nm. Glycogen was determined at 620 nm using microspectrometer.

### Cell culture and bacterial culture

A rat H9c2 cardiomyocyte cell line, obtained from American Type Cultural Collection (CRL1446, ATCC, USA), was cultured in Dulbecco's modified Eagle's medium (DMEM, Gibco, Oklahoma, USA) supplemented with 10% fetal bovine serum (FBS, Gibco, Oklahoma, USA) and 1% v/v penicillin/streptomycin (Gibco, Oklahoma, USA) at 37°C in a 5% CO_2_ humidity environment. *Desulfovibrio vulgaris* was cultured in Brain Heart Infusion (OXOID, England) broth in the DWS A25 anaerobic chamber (Whitely, England) for 24 h. The metabolic products of *D. vulgaris* (MPDV) in the culture medium were centrifuged at 5,000 rpm to collect the supernatant for further experiments.

### Evaluation of mitochondrial morphology

H9c2 cells were seeded into μ-Slide 8-well glass bottom plate (#80826, ibidi, Germany) at a total number of 10,000 per well. After treatments of MPDV (0, 10, 20, 40%), cells were incubated with 100 nM MitoTracker Red CMXRos (Beyotime, China) at 37°C for 20 min. Then cells were washed using Dulbecco's Modified Eagle Medium (DMEM) for five times. The images of mitochondrial morphology were captured by confocal microscopy (LSM 800, ZEISS, USA) equipped with a 63× oil immersion objective. Red fluorescence represented mitochondria stained by MitoTracker Red CMXRos.

### Mitochondrial membrane potential determination

H9c2 cells were seeded into μ-Slide 8-well glass bottom plate (#80826, ibidi, Germany) at a total number of 1 × 10^4^ per well. To measure the mitochondrial membrane potential (MMP), JC-1 mitochondrial membrane potential assay kit (Beyotime, China) was used according to the manufacturer's protocol. After treatments of MPDV (0, 10, 20, 40%), cells were incubated with 10 μg/ml JC-1 at 37°C for 20 min. The fluorescent images of JC-1 were captured by red and green fluorescence, respectively with Confocal Microscopy (LSM 800, ZEISS, USA) equipped with a 63× oil immersion objective. For the quantification of MMP, H9c2 cells were seeded in 96 well black/clear bottom plates. Ex 488/Em 535 nm and Ex 550/Em 600 nm were used and MMP was calculated by the ratio of red to green fluorescence.

### Reactive oxygen species determination

H9c2 cells were seeded into μ-Slide 8-well glass bottom plate (#80826, ibidi, Germany) at a total number of 1 × 10^4^ per well. To measure the Reactive Oxygen Species (ROS) level, DCFH-DA ROS assay kit (Beyotime, China) was used according to the manufacturer's protocol. After treatments of MPDV (0, 10, 20, 40%), cells were incubated with 5 μM DCFH-DA at 37°C for 30 min. The fluorescent images of DCFH-DA were captured by green fluorescence with Confocal Microscopy (LSM 800, ZEISS, USA) equipped with a 63× oil immersion objective.

### ATP Assay

The ATP level of H9c2 cells, after treatments of MPDV (0, 10, 20, 40%), was measured by using a luminescent ATP assay kit (Beyotime, China) according to the manufacturer's protocol. The levels of ATP were detected by the Multi-Mode Detection Platform and calculated as the ratio to the control group (set as 1.0), respectively.

### Calcium determination

H9c2 cells were seeded into μ-Slide 8-well glass bottom plate (#80826, ibidi, Germany) at a total number of 1 × 10^4^ per well. To measure the calcium (Ca^2+^) ion level, Fluo-4 AM calcium assay kit (Beyotime, China) was used according to the manufacturer's protocol. After treatments of MPDV (0, 10, 20, 40%), cells were incubated with 2 μM Fluo-4 AM probe at 37°C for 30 min. The fluorescent images of Fluo-4 AM were captured by green fluorescence with confocal microscopy (LSM 800, ZEISS, USA) equipped with a 63× oil immersion objective.

### Cell viability assay

Cytotoxicity was assessed by cell viability assay. Cell viability was measured by using 3-(4,5-dimethylthiazol-2-yl)-2,5-diphenyltetrazolium bromide assay (MTT assay kit, Beyotime, China). The absorbance of 570 nm was evaluated by a Multi-Mode Detection Platform (SpectraMax Paradigm, Molecular Devices, USA). Cell viability was calculated as the ratio to the control group (set as 1.0), respectively.

### Statistical analysis

The functional prediction of GM was performed using FAPROTAX on the Cloud-platform of Biomicroclass (http://www.cloud.biomicroclass.com/CloudPlatform). SPSS version 22.0 and GraphPad prism version 9.0 were used for statistical analysis and graphical presentation. Kolmogorov–Smirnov test was used to determine the data normality. Student *t*-test, Kruskal-Wallis (non-parametric data) tests, and Two-way ANOVA (for parametric data) were employed to determine the significant changes. Dunn-Bonferroni test and Bonferroni test were performed for parametric and non-parametric multiple comparison *p*-values correction, respectively.

## Results

### HA treatment altered GM profile of the mice assessed by ERIC-PCR

To investigate the effect of HA on the mice's GM profile, 6–8 weeks old C57BL/6 mice were orally fed daily with 50 mg/kg HA for 15 days. Fecal samples were collected on Days 0 and 15 from individual mice for gut microbial DNA extraction (Figure 1A). After 15 days of treatment with HA, a slight decrease in food consumption and body weight were detected in the HA group compared with the control group, but the differences are not statistically significant. Comparing the body weight of the Ctrl vs. HA groups, the *P*-values for 0-, 5-, 10-, and 15-day were >0.9999, 0.2284, 0.6583, and 0.2386, respectively) (Figure 1B). ERIC-PCR and gel electrophoresis were performed as previously described (25). The resulting data were plotted with PLS-DA to display the similarity of GM profiles between the HA-treated and the control mice. Results showed a similar GM composition between the control and HA groups on day 0. However, by the end of the experiment, HA groups displayed an obvious different GM profile compared with the control group (Figure 1C; Supplementary Figures S1, S2).

### HA altered the GM diversity and composition

**Figure 2:**
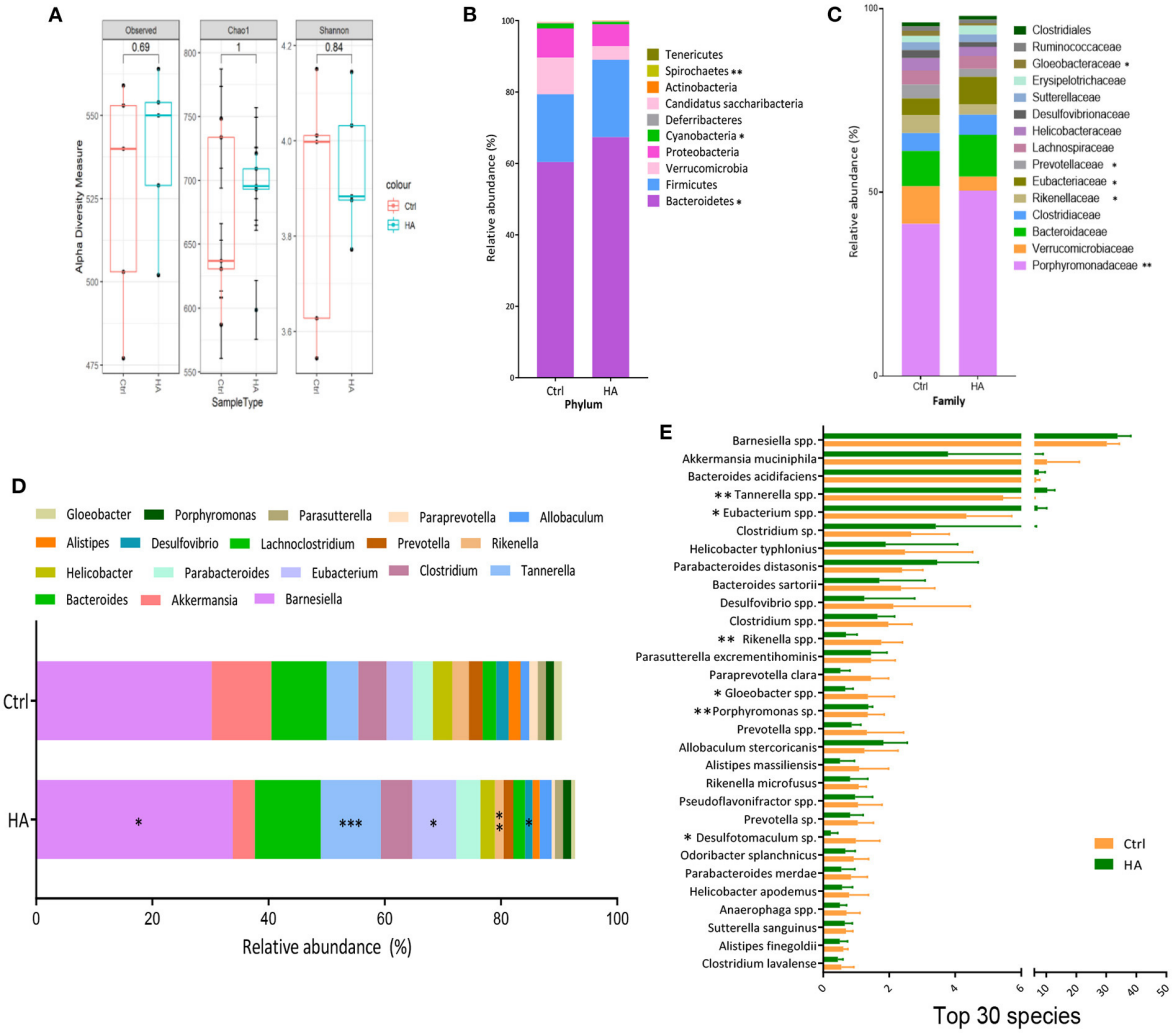
HA changed the core GM composition. **(A)** Alpha diversity analysis of the GM compositions between the control and the HA groups. **(B)** The relative abundance of the dominant phyla. **(C)** The relative abundance of the dominant family. **(D)** The relative abundance of the dominant genus. **(E)** The relative abundance of the Top 30 species. *n* = 5. *p < 0.05; **p < 0.01; ***p < 0.001.

To further analyze the change of GM induced by treatment with HA, 16S rRNA gene sequencing was used to profile the GM composition from fecal genomic DNA. The alpha diversity analysis showed that HA treatment decreased the alpha diversity (observed and Chao1 indexes) and richness (Shannon index) compared with the control group (Figure 2A). Compared with the control group, HA treatment significantly altered the relative abundance of the phyla Bacteroidetes, Cyanobacteria, and Spirochaetes (Figure 2B). Meanwhile, in family taxa, HA mainly increased Porphyromonadaceae, Eubacteriaceae, and decreased Verrucomicrobioacease, Rikenellaceae, Prevotellaceae, Gloeobacteraceae (Figure 2C). Moreover, in genus taxa, HA significantly increased the relative abundance of *Barnesiella, Tannerella*, and *Eubacterium*, while reduced *Rikenella* and *Desulfovibrio* (Figure 2D). The change of GM genus taxa was associated with the increase of species *Barnesiella* spp., *Tannerella* spp., and *Eubacterium* spp., whereas the decrease of *Rikenella* spp. and *Desulfovibrio* sp. (Figure 2E).

### HA decreased the relative abundance of potential pathogens and increased the beneficial bacteria

**Figure 3:**
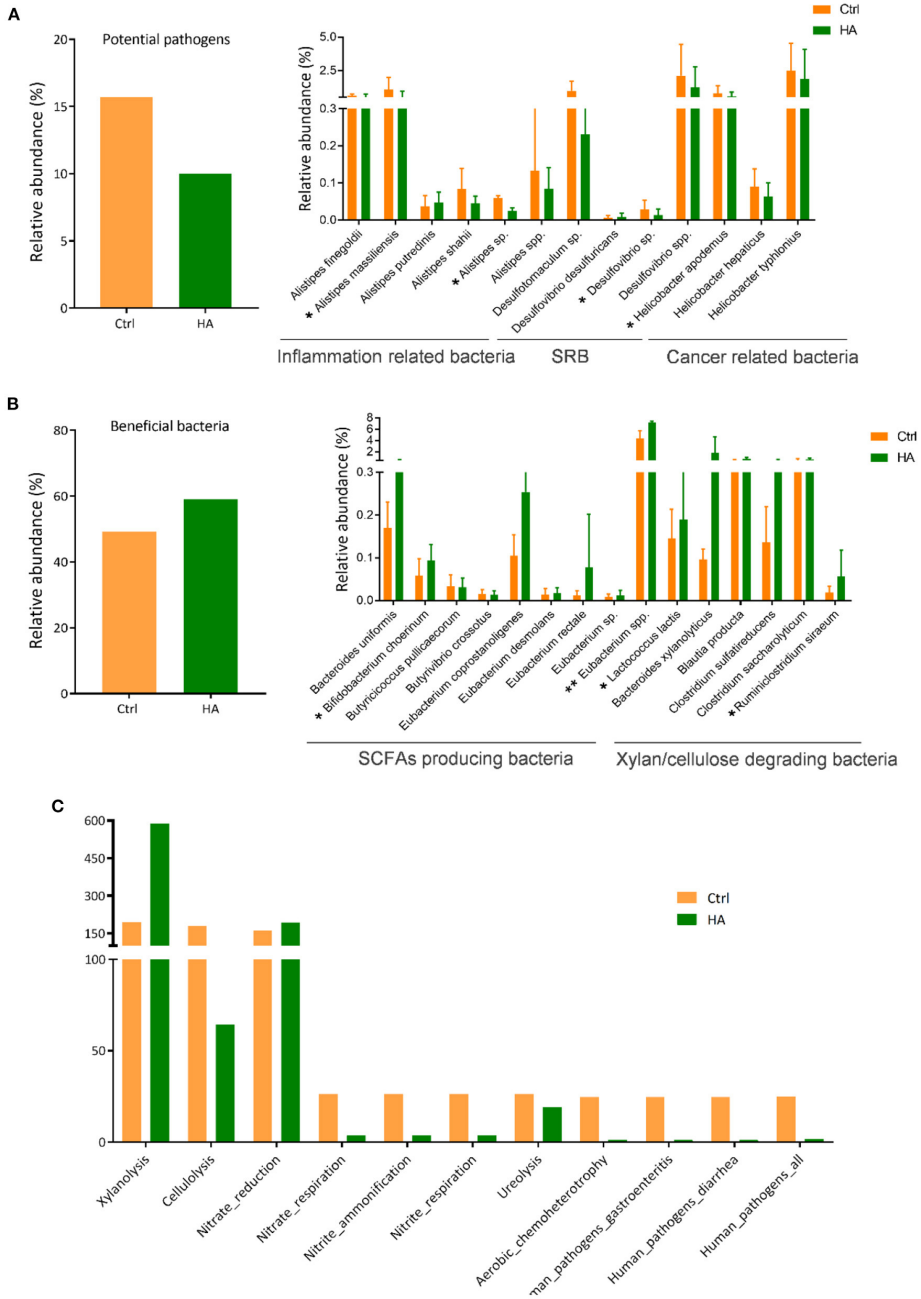
HA altered the relative abundance of the potential pathogens and beneficial bacteria in the experimental mice. **(A)** The total OTU of potential pathogens of the control and HA-treated mice. **(B)** The total OTU of beneficial bacteria of the control and HA-treated mice. SRB, sulfate-reducing bacteria. SCFAs, short-chain fatty acids. **(C)** The results of FAPROTAX functional prediction of GM in the control and HA groups. SRB, sulfate-reducing bacteria. *n* = 5. *p < 0.05; **p < 0.01.

Based on the published literature from the NCBI database (PubMed), we grouped the altered species into potential pathogenic and beneficial bacteria (Supplementary Tables S1, S2). The potential pathogens suppressed by HA treatment were mainly the species from the genus *Alistipes* associated with inflammation, the sulfate-reducing bacteria (SRB) *Desulfovibria*, and the *Helicobacter* associated with gastrointestinal inflammation and cancer (Figure 3A).

On the other hand, HA could promote the growth of certain beneficial bacteria, especially the short-chain fatty acids (SCFAs)-producing bacteria and xylan/cellulose-degrading bacteria (Figure 3B). Among the SCFAs-producers are *Bacteroides uniformis, Bifidobacterium cheorinum, Eubacterium rectale*, and *Eubacterium* spp. The SCFAs-producers play an important role to break down indigestible foods and convert to acetate, propionate, butyrate, and valerate for gut-healthy maintenance (30). Meanwhile, HA treatment also stimulated the growth of xylan/cellulose degrading bacteria, including *Bacteroides xylanolyticus, Blautia producta*, and *Clostridium sulfatireducens*.

Furthermore, FAPROTAX was used to evaluate the impact of HA on GM metabolic pathways. The findings showed that HA increased the GM xylanolysis function and nitrate reduction. Simultaneously, HA reduced the accumulation of nitrite respiration and nitrate ammonification. More importantly, results also showed that HA could reduce the accumulation of the pathogens-associated functional pathways, including gastroenteritis and diarrhea (Figure 3C).

### HA revealed anti-fatigue effects by modulating the levels of SOD, MDA, and glycogen in mice's serum, liver, and muscle

**Figure 4:**
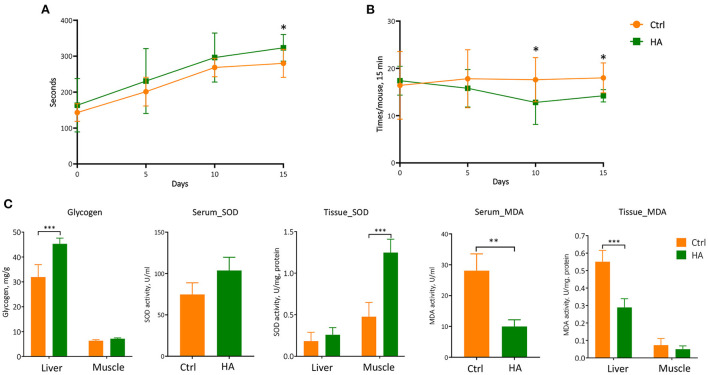
The anti-fatigue property of HA. **(A)** The retention time on the rotarod of the mice from three consecutive attempts. **(B)** The times of fall of the mice within 15 min. **(C)** The level of the glycogen, SOD, and MDA in the serum, liver, and muscle. *n* = 5. *p < 0.05; **p < 0.01; ***p < 0.001.

The above positive modulating effects on GM triggered our investigation of the health status of the HA-treated mice. We first took on the rotarod test to evaluate the potential anti-fatigue property of HA. C57BL/6 mice at 6–8 weeks of age were first trained on the rotarod for 5 min. After 30 min rest, mice were tested on the rotarod for 15 min each time for three consecutive trials for each mouse. The retention time on the rotarod from all three attempts was recorded and analyzed. The results showed that HA-treated mice significantly yielded higher scores on the retention time than the untreated control (Figure 4A). Moreover, the results also showed that HA-treated mice fell from the rotarod less than the control mice (Figure 4B). The fatigue-associated biomarkers, SOD, MDA, and glycogen were measured in the serum, liver, and muscle samples. The results showed that HA significantly increased the level of glycogen in the liver. Meanwhile, the levels of SOD in the serum and muscle were effectively increased in the HA group. While the level of MDA in the serum and liver was markedly decreased after 15 days of HA treatment compared to the control group (Figure 4C).

### The metabolic products of *Desulfovibrio vulgaris* caused damage to mitochondrial functions

**Figure 5:**
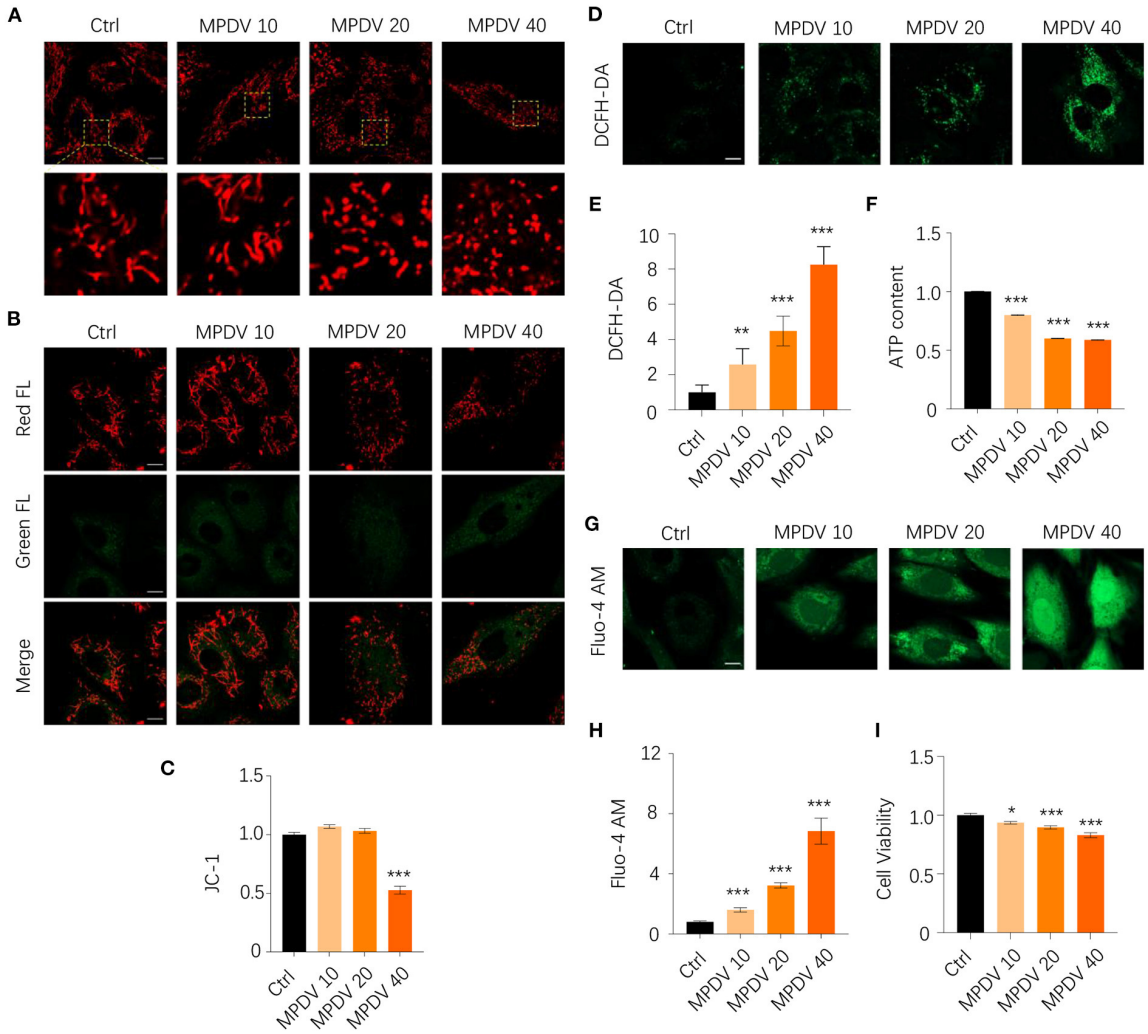
MPDV treatment is detrimental to mitochondrial biological functions and cell viability. **(A)** H9c2 cardiomyocytes were treated with MPDV (0, 10, 20, and 40%) for 24 h. Mitochondrial morphology was observed by Zeiss 800 confocal microscopy with 63× oil objective by using Mitotracker staining dye with red fluorescence. Magnified photographs showed a detailed view of the yellow area indicated in the upper panels. **(B,C)** Mitochondrial membrane potential was observed by Zeiss 800 confocal microscopy with 63× oil objective by using JC-1 staining dye with both red and green fluorescence. The ratio of red/green FL was calculated by the fluorescence microplate assay. **(D,E)** ROS level was observed by Zeiss 800 confocal microscopy with 63× oil objective by using DCFH-DA staining dye. The green FL was calculated from different views. **(F)** ATP production is detected by the luminescent detection assay in 96 well white/clear bottom plates. **(G,H)** Calcium level was observed by Zeiss 800 confocal microscopy with a 63× oil objective using DCFH-DA staining dye. The green FL was calculated from different views. **(I)** Cell viability is detected by MTT assay. Data (*n* = 6) were shown as the mean ± SEM (*p < 0.05, **p < 0.01,***p < 0.001). Scale bar: 10 μm.

As mentioned above, HA reduced the relative abundance of *Desulfoviibrio* spp. in HA-treated mice. We wonder whether the metabolic products of this group of bacteria might have harmful effects on mitochondrial functions. We then chose *D. vulgaris*, the representative *Desulfovibrio* bacteria for mitochondria functional tests in cardiomyocytes. We firstly used mitotracker staining dye to evaluate the mitochondrial morphology exposed to MPDV at the dosages of 0, 10, 20, and 40% in H9c2 cardiomyocytes. Under normal conditions, mitochondria displayed thread-like or punctate morphology due to their dynamic fusion-fission balance. However, MPDV treatment significantly changed mitochondrial morphology, contributing to punctate morphology to a large extent. The presence of MPDV caused mitochondrial fragmentation in a dose-dependent manner (Figure 5A). Mitochondrial membrane potential (ΔΨm) is a sensitive and important index to evaluate mitochondrial health. MPDV (40%) treatment significantly caused the decrease of ΔΨm when compared with the control group by using JC-1 staining dye, suggesting MPDV induced mitochondrial depolarization (Figures 5B,C). As mitochondrial membrane potential was an important factor to generate ATP along the electron transport chain (ETC), decreasing ΔΨm could lead to excessive ROS generation. Application of DCFH-DA, a ROS-specific staining dye, showed that MPDV treatment enhanced ROS burden in a dose-dependent manner (Figures 5D,E). Furthermore, the decrease of ΔΨm probably caused the reduction of ATP. Therefore, the assessment of ATP content was used to evaluate MPDV in mitochondrial biological functions. The luminescent assay showed that MPDV decreased the ATP content in a dosage-dependent manner (Figure 5F). we also evaluated the Ca^2+^ level in treated cells since defects of mitochondrial functions might trigger mitochondrial and cytosolic Ca^2+^ overloading. Therefore, we applied Fluo-4 AM staining dye to measure Ca^2+^ level. The result showed that strong signals of Ca^2+^ were detected after MPDV treatment in the whole cell (Figures 5G,H). Finally, MPDV also decreased cell viability, suggesting its cytotoxic effects (Figure 5I). Taken together, the presence of MPDV could damage the mitochondrial functions and exert cytotoxic effects on H9c2 cardiomyocytes.

## Discussion

HA is widely applied in the commercial field, such as skin care, cosmetic products, eye drops, and health supplements for various medicinal purposes (20, 31, 32). However, the physiological effect of the ingested HA is unclear. In this study, we firstly reported the prebiotic effects of HA in C57BL/6 mice. Secondly, we revealed the anti-fatigue effects by monitoring the level of glycogen, SOD, and MDA in serum, liver, and muscle. Lastly, we demonstrated that the sulfate-reducing bacteria, which was suppressed by the treatment of HA, could have detrimental effects on mitochondrial functions.

Fibers, chitin, and polysaccharides are the common prebiotics possessing many bioactivities to benefit the host (12, 13, 33, 34). HA is an important constituent of animals. It is not hard to understand the demand for HA as medicinal products and food supplements. As some of the HA supplements are ingested and digested in the gut, it is important to investigate the impact of HA on GM and the potential biological consequence of the HA and GM interaction. This study demonstrated that HA specifically reduced several groups of pathogenic gut microbes, such as *Alistipes*, SRB, and *Helicobacter*, which are usually associated with gut inflammation and cancer (35–37). Moreover, our results also showed that HA could increase the relative abundance of certain beneficial bacteria, including the SCFAs-producing bacteria and xylan/cellulose-degrading bacteria. These bacteria could provide a series of benefits for the host through their metabolites. SCFAs, the end products of fermented dietary fibers by the gut microbes, are indispensable in maintaining colon health and act as an energy source for the growth and renewal of colonocytes (38, 39). Reports showed that butyrate could maintain the homeostasis of the host immune system and possess anti-inflammatory and anti-cancer effects (40, 41).

Fatigue is a complex metabolic phenomenon that influences physical performance. Fatigue could induce changes in performance that leads to decreasing muscle power and endurance, as well as decreasing the motor skill performance and diminishing the physical and mental functions. The mechanism underlying fatigue is extremely complex, such as energy metabolism disorder and oxidative stress (42). Glycogen, SOD, and MDA are the main biomarkers to evaluate the effects of anti-fatigue agents. Glycogen is the main storage form of glucose and can be quickly metabolized to glucose to meet the sudden need for energy (43). Depletion of glycogen leads to a reduction in ATP regeneration and impairs the contractile activity of the muscle (44). Studies showed a significant decrease of glycogen in both liver and muscle when the body is fatigued, however, increasing the level of glycogen could attenuate the condition of fatigue (23, 45). The balance of oxidation and anti-oxidation in the body is usually disrupted by exhaustive exercise. When the body was fallen into the condition of oxidative stress and reactive oxygen species, it would evoke fatigue. MDA is a product of both lipid peroxidation and prostaglandin biosynthesis. The level of MDA is the sensitive indicator of the metabolism of free radicals which could reflect the severity of the oxidative reaction. SOD is also an enzyme that plays a key role in maintaining the balance of oxidation and anti-oxidation. SOD can remove hydroxyl products that were produced by the physiological process to protect the surrounding cells from damage (46). Reports also showed that increasing the level of SOD could help to improve the body's anti-fatigue (47, 48). Here, glycogen, SOD, and MDA were selected to evaluate the anti-fatigue effects of HA in the mouse model. And our data showed that HA revealed a strong anti-fatigue effect by increasing the level of glycogen and SOD, but decreasing the level of MDA in serum, liver, and muscle.

ATP is the direct source of energy. Mitochondria is the crucial place for ATP synthesis and metabolism. *D. vulgaris* is a model organism for studying the energy metabolism of SRB (49). SRB is found in gut-associated diseases, including inflammatory bowel disease, irritable bowel syndrome, celiac disease, and colorectal cancer (50). The pathogenesis of SRB is the metabolic product, hydrogen sulfide (H2S) that could cause DNA damage and intestinal inflammation (51–53). Here, for the first time, we found that the metabolic products of *D. vulgaris* were detrimental to mitochondrial biological functions (including ATP synthesis) and cell viability. Notably, the treatment of HA showed an obvious reduction in the relative abundance of SRB in the mice model. These results indicated that inhibition of SRB growth was probably another anti-fatigue mechanism of HA.

Collectively, in this study, our results showed that HA possessed prebiotic effects by increasing the relative abundance of SCFAs-producing bacteria and xylan-degrading bacteria, while reducing potential pathogens, including SRB, inflammation-, and cancer-associated bacteria. Moreover, our results also showed that HA revealed anti-fatigue properties *via* increasing the level of glycogen and SOD, however, decreasing the level of MDA in serum, liver, and muscle. Additionally, HA could exhibit anti-fatigue effects by reducing the growth of SRB which was detrimental to mitochondrial biological functions (including ATP synthesis) and cell viability.

## Data availability statement

The datasets presented in this study can be found in online repositories. The names of the repository/repositories and accession number(s) can be found below: NCBI SRA; PRJNA855021.

## Ethics statement

The animal study was reviewed and approved by the animal welfare and experiments strictly followed the procedures approved by the Ethics Review Committee of Macau University of Science and Technology. Written informed consent was obtained from the owners for the participation of their animals in this study.

## Author contributions

GH conducted the experiments, analyzed sequencing data, and composed the manuscript. LS carried out the anti-fatigue experiment and wrote the results of the associated research. NZ and XR designed and carried out experiments on mitochondria and ATP synthesis and composed their results. RH, WL, and XL carried out animal experiments on the prebiotic effects of HA. WH coordinated and supervised the project and revised the manuscript. All authors contributed to the article and approved the submitted version.

## Funding

This work was supported by the National Nature Science Foundation of China (Grant Nos. 31900103 and 31900498) and the Postdoctoral Science Foundation of China (Grant No. 2019M662037). This work was also supported by the Science and Technology Development Fund of the Macau Government (Grant No. FDCT 0054-2018-A2) and the Specialized Research Fund for the Technology Innovation of Foshan City, Foshan, China.

## Conflict of interest

The authors declare that the research was conducted in the absence of any commercial or financial relationships that could be construed as a potential conflict of interest.

The handling editor IK declared a past collaboration with the authors GH and WH.

## Publisher's note

All claims expressed in this article are solely those of the authors and do not necessarily represent those of their affiliated organizations, or those of the publisher, the editors and the reviewers. Any product that may be evaluated in this article, or claim that may be made by its manufacturer, is not guaranteed or endorsed by the publisher.
