# Supplementary material for: The prebiotic and anti-fatigue effects of hyaluronan
Source: Front Nutr. 2022 Aug 8;9:977556. doi: 10.3389/fnut.2022.977556 (PMC9393540; doi:10.3389/fnut.2022.977556)

**The prebiotic and anti-fatigue effects of hyaluronan**

Guoxin Huang^1,2,3#^, Lu Su^1#^, Ni Zhang^4,5^, Ruixuan Han^1^, Wai Kit Leong^1^, Xiaoang Li^1^, Xuecong Ren^6*^, W.L. Wendy Hsiao^1,7*^

1. State Key Laboratory of Quality Research in Chinese Medicine, Macau University of Science and Technology, Macau, China

2. Clinical Research Center, Shantou Central Hospital, Shantou, China

3. Zhuhai MUST Science and Technology Research Institute, Zhuhai, China

4. Department of Cell Biology, Zhejiang University School of Medicine, Hangzhou, Zhejiang, China.

5. Zhejiang University Medical Center, Hangzhou, Zhejiang, China.

6. Center for Stem Cell and Regenerative Medicine, Zhejiang University School of Medicine, Hangzhou, China

7. Foshan Women and Children Hospital Affiliated with Southern Medical University, Foshan, China

# Authors equally contributed to this work.

* Correspondence should be addressed to W. L. Wendy Hsiao, bowhsiao@gmail.com and Xuecong Ren, [renxc@zju.edu.cn](mailto:renxc@zju.edu.cn).

**Table S1. The list of potential beneficial bacteria modulated by HA treatment**

| **Species** | **Ctrl (Mean±SD)** | **HA (Mean±SD)** | **Fold change (%)** |
| --- | --- | --- | --- |
| Anaerostipes butyraticus | 0.01±0.01 | 0.02±0.03 | 77.93 |
| Anaerostipes caccae | 0.01±0.01 | 0.01±0.02 | 100.78 |
| Anaerotruncus colihominis | 0.01±0.01 | 0.01±0.01 | -14.25 |
| Bacteroides acidifaciens | 6.66±1.04 | 7.51±1.86 | 12.84 |
| Bacteroides sartorii | 2.36±0.91 | 1.70±1.24 | -27.82 |
| Bacteroides spp. | 0.23±0.26 | 0.04±0.01 | -82.47 |
| Bacteroides uniformis | 0.17±0.05 | 0.31±0.23 | 85.26 |
| Bacteroides xylanisolvens | 0.00±0.00 | 0.01±0.00 | 698.30 |
| Bacteroides xylanolyticus | 0.10±0.02 | 1.78±2.56 | 1754.01 |
| Barnesiella spp. | 30.18±3.72 | 33.77±3.93 | 11.88 |
| Bifidobacterium choerinum | 0.06±0.04 | 0.09±0.03 | 59.72 |
| Blautia producta | 0.43±0.17 | 0.70±0.23 | 64.29 |
| Brucella sp. | 0.10±0.09 | 0.03±0.02 | -71.79 |
| Butyricicoccus pullicaecorum | 0.03±0.02 | 0.03±0.02 | -6.37 |
| Butyrivibrio crossotus | 0.02±0.01 | 0.01±0.01 | -13.67 |
| Clostridium sulfatireducens | 0.14±0.07 | 0.31±0.30 | 130.49 |
| Eubacterium coprostanoligenes | 0.10±0.04 | 0.25±0.11 | 142.11 |
| Eubacterium desmolans | 0.01±0.01 | 0.02±0.01 | 23.57 |
| Eubacterium oxidoreducens | 0.00±0.00 | 0.00±0.00 | 0.12 |
| Eubacterium rectale | 0.01±0.01 | 0.08±0.11 | 527.10 |
| Eubacterium ruminantium | 0.00±0.00 | 0.00±0.00 | -20.19 |
| Eubacterium sp. | 0.01±0.01 | 0.01±0.01 | 50.14 |
| Eubacterium spp. | 4.34±1.23 | 7.16±2.65 | 64.95 |
| Lachnoclostridium clostridium saccharolyticum | 0.46±0.29 | 0.65±0.19 | 39.62 |
| Lachnoclostridium clostridium scindens | 0.02±0.02 | 0.02±0.03 | -8.50 |
| Lachnoclostridium clostridium xylanolyticum | 0.02±0.01 | 0.05±0.07 | 168.12 |
| Lachnoclostridium eubacterium contortum | 0.22±0.09 | 0.17±0.09 | -24.35 |
| Lactobacillus gasseri | 0.20±0.11 | 0.13±0.05 | -33.06 |
| Lactobacillus intestinalis | 0.03±0.01 | 0.01±0.01 | -47.34 |
| Lactobacillus reuteri | 0.06±0.02 | 0.05±0.03 | -7.17 |
| Lactococcus lactis | 0.15±0.06 | 0.19±0.10 | 30.15 |
| Parabacteroides distasonis | 2.39±0.56 | 3.46±1.11 | 44.59 |
| Ruminiclostridium [eubacterium] siraeum | 0.02±0.01 | 0.06±0.05 | 207.09 |
| Ruminiclostridium clostridium cellobioparum | 0.04±0.02 | 0.04±0.01 | 5.05 |
| Ruminiclostridium clostridium leptum | 0.10±0.07 | 0.05±0.02 | -49.39 |
| Ruminiclostridium eubacterium siraeum | 0.05±0.04 | 0.02±0.01 | -62.84 |
| Ruminococcus flavefaciens | 0.22±0.16 | 0.12±0.04 | -45.01 |
| Ruminococcus sp. | 0.16±0.10 | 0.12±0.05 | -24.75 |
| Tyzzerella clostridium colinum | 0.01±0.01 | 0.03±0.02 | 109.34 |

**Table S2. The list of potential pathogens modulated by HA treatment**

| **Species** | **Ctrl (Mean±SD)** | **HA (Mean±SD)** | **Fold change (%)** |
| --- | --- | --- | --- |
| *Alistipes finegoldii* | 0.61±0.12 | 0.50±0.21 | -17.97 |
| *Alistipes massiliensis* | 1.08±0.80 | 0.51±0.39 | -53.31 |
| *Alistipes putredinis* | 0.04±0.03 | 0.05±0.03 | 27.71 |
| *Alistipes shahii* | 0.08±0.05 | 0.04±0.02 | -47.14 |
| *Alistipes sp.* | 0.06±0.01 | 0.02±0.01 | -58.60 |
| *Alistipes spp.* | 0.13±0.20 | 0.09±0.05 | -36.49 |
| *Anaeroplasma spp.* | 0.01±0.01 | 0.00±0.00 | -73.68 |
| *Barnesiella intestinihominis* | 0.03±0.01 | 0.06±0.03 | 80.57 |
| *Citrobacter spp.* | 0.02±0.02 | 0.01±0.01 | -62.61 |
| *Clostridium spp.* | 1.97±0.64 | 1.64±0.47 | -16.66 |
| *Desulfotomaculum sp.* | 0.99±0.65 | 0.23±0.19 | -76.48 |
| *Desulfovibrio desulfuricans* | 0.01±0.01 | 0.01±0.01 | 44.49 |
| *Desulfovibrio sp.* | 0.03±0.02 | 0.01±0.01 | -52.42 |
| *Desulfovibrio spp.* | 2.12±2.09 | 1.24±1.36 | -41.39 |
| *Escherichia coli* | 0.08±0.14 | 0.00±0.00 | -95.94 |
| *Eubacterium plexicaudatum* | 0.05±0.04 | 0.02±0.01 | -57.81 |
| *Helicobacter apodemus* | 0.79±0.53 | 0.57±0.28 | -27.27 |
| *Helicobacter hepaticus* | 0.09±0.04 | 0.06±0.03 | -29.05 |
| *Helicobacter typhlonius* | 2.48±1.83 | 1.89±1.95 | -23.59 |
| *Lachnoclostridium clostridium hathewayi* | 0.07±0.07 | 0.04±0.03 | -39.12 |
| *Lachnoclostridium clostridium indolis* | 0.37±0.33 | 0.32±0.28 | -13.59 |
| *Oscillospira spp.* | 0.41±0.29 | 0.29±0.28 | -29.43 |
| *Parabacteroides goldsteinii* | 0.18±0.13 | 0.14±0.03 | -23.50 |
| *Parabacteroides merdae* | 0.85±0.44 | 0.55±0.37 | -35.11 |
| *Prevotella sp.* | 1.05±0.43 | 0.82±0.35 | -21.91 |
| *Rikenella spp.* | 1.76±0.57 | 0.69±0.30 | -60.80 |
| *Robinsoniella peoriensis* | 0.33±0.26 | 0.18±0.12 | -47.14 |

**Figure S1:** The analysis of similarity of ERIC-PCR on day 0 using AONSIM.

**ANOSIM statistic R:** 0.1444

**P-value:** 0.152 (Not significant)


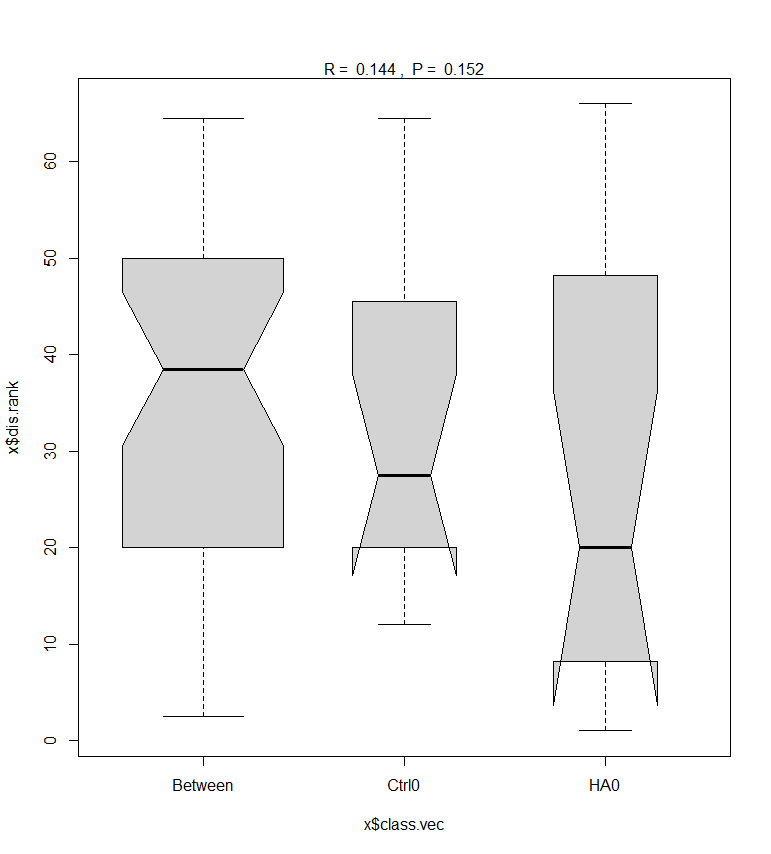


**Figure S2:** The analysis of similarity of ERIC-PCR on day 0 using AONSIM.

**ANOSIM statistic R:** 0.3148

**P-value:** 0.024 (Significant)


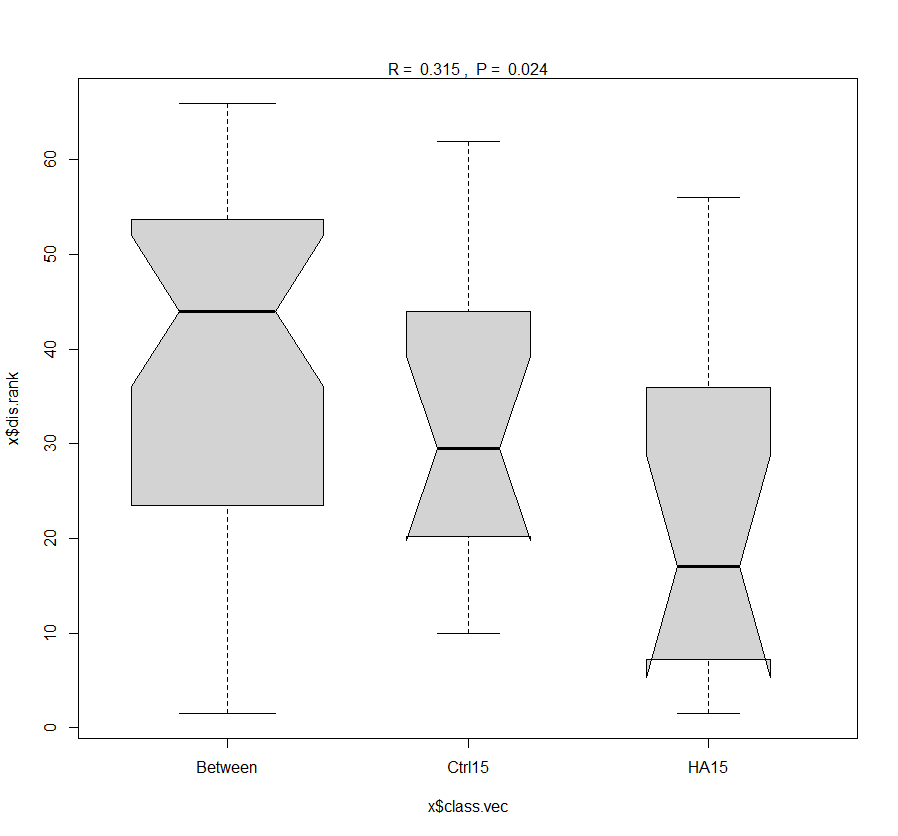

Supplement: Supplementary file 1 [file Data_Sheet_1.docx]
